# Modelling the impacts of pests and diseases on agricultural systems

**DOI:** 10.1016/j.agsy.2017.01.019

**Published:** 2017-07

**Authors:** M. Donatelli, R.D. Magarey, S. Bregaglio, L. Willocquet, J.P.M. Whish, S. Savary

**Affiliations:** a CREA - Council for Agricultural Research and Economics, Research Center for Agriculture and Environment, via di Corticella 133, I-40128, Bologna, Italy; b Center for Integrated Pest Management, North Carolina State University, Raleigh, NC 27606, USA; c AGIR, Université de Toulouse, INRA, INPT, INP- EI PURPAN, Castanet-Tolosan, France; d CSIRO Agriculture and Food, 203 Tor St Toowoomba, Qld 4350, Australia

**Keywords:** Model coupling, Model integration, Process-based models, Yield loss, Modelling frameworks

## Abstract

The improvement and application of pest and disease models to analyse and predict yield losses including those due to climate change is still a challenge for the scientific community. Applied modelling of crop diseases and pests has mostly targeted the development of support capabilities to schedule scouting or pesticide applications. There is a need for research to both broaden the scope and evaluate the capabilities of pest and disease models. Key research questions not only involve the assessment of the potential effects of climate change on known pathosystems, but also on new pathogens which could alter the (still incompletely documented) impacts of pests and diseases on agricultural systems. Yield loss data collected in various current environments may no longer represent a adequate reference to develop tactical, decision-oriented, models for plant diseases and pests and their impacts, because of the ongoing changes in climate patterns. Process-based agricultural simulation modelling, on the other hand, appears to represent a viable methodology to estimate the impacts of these potential effects. A new generation of tools based on state-of-the-art knowledge and technologies is needed to allow systems analysis including key processes and their dynamics over appropriate suitable range of environmental variables. This paper offers a brief overview of the current state of development in coupling pest and disease models to crop models, and discusses technical and scientific challenges. We propose a five-stage roadmap to improve the simulation of the impacts caused by plant diseases and pests; i) improve the quality and availability of data for model inputs; ii) improve the quality and availability of data for model evaluation; iii) improve the integration with crop models; iv) improve the processes for model evaluation; and v) develop a community of plant pest and disease modelers.

## Highlights

• Overview of the current state of development in coupling pest and disease models to crop models
• Technical and scientific challenges in coupling pest and disease model to crop models
• Roadmap to improve the simulation of the impacts caused by plant diseases and pests

## 1 Introduction

Quantifying the impacts of plant pests and diseases on crop performances represents one of the most important research questions for agricultural simulation modelling (Newman et al., 2003, Savary et al., 2006, Esker et al., 2012, Whish et al., 2015a). In the past, theoretical frameworks were thus developed to take into account the impact of pests and disease on yield as separated by the other limiting factors due to genotype x environment x management interactions. De Wit and Penning de Vries (1982) introduced the concept of production situation, which encompasses the combination of yield defining and yield limiting factors, therefore determining the attainable yield. A production situation also includes farmer crop management including pest and disease management. This widely accepted categorization of yield levels incorporates the crop genetics among the factors defining potential yield, and groups the water and nitrogen stress as limiting factors to attainable yield. Later, Rabbinge (1993) defined (1) a potential yield, defined by solar radiation and temperature, (2), the attainable yield, limited by water and nutrient availability, and (3) the actual yield, reduced by diseases, pests, and environmental stressors. According to this framework, reduction of crop yield due to biotic stresses corresponds to the difference between the attainable and actual yield.

The classification of yield levels constitutes the basis to guide strategic decisions in the development and application of cropping system models (e.g., Jagtap et al., 1999, Cheero-Nayamuth et al., 2000, Abeledo et al., 2008), including the quantification and modelling of yield losses (Zadoks and Schein, 1979, Savary et al., 2006, Esker et al., 2012). For instance, a common procedure in the calibration of cropping system models is to simulate the attainable yield, that is, the yield of an uninjured (disease and pest free) crop. These models are parameterized by comparing model outputs with reference data collected in experimental trials where there is little or no biophysical stress, so that yields are close to potential production. This reduces the impact of experimental noise on the parameters representing the crop morpho-physiological traits (Wolf and de Wit, 2010, Djabi et al., 2013, Bregaglio et al., 2015). Also, most of the available crop system models offer options that enable the user decide to activate nutrients and water limitation, with a default configuration running the potential production level (e.g., WOFOST, Supit et al., 1994, Boogaard et al., 2011,; DSSAT, Jones et al., 2003, CropSyst, Stöckle et al., 2003; AquaCrop, Raes et al., 2009). Currently, such a “pest and disease switch” is still missing in many crop models, although developments in the last decades are moving towards the quantitative description of the impact of pest and diseases on yield.

Plant pathogens and crop-feeding insects are integral part of agroecosystems, where they have coevolved with crops over millennia (McCann et al., 2013). A cascade of mutual and complex interactions exists between the cultivated crops and their pests and diseases (Berger et al., 2007). Two main groups of processes may be considered to address these systems, corresponding to scientific domains where modelling, in very diverse forms, has developed. A first group is related to pathogen population dynamics, and concerns the dynamics of Pests and Disease Models (PDM), through which populations may spatially expand and temporally increase. The second group addresses crop losses, and focuses on the consequences of the host-pathogen interactions on crop physiological processes and yield. These two broad groups of processes are strongly responsive to physical, biological, social, and economic factors where crops are cultivated (Zadoks and Schein, 1979). These two scientific domains were recently discussed by Cunniffe et al. (2015), who identified the linking of epidemiological models to yield and ecosystem services as the first challenge in modelling plant disease, stating that models should incorporate sufficient *epidemiological realism* in order to analyse and predict the *effects of disease and host dynamics on yield*.

Additional key research questions involve the assessment of the potential effects of climate change (Rosenzweig et al., 2001), of technology shifts (Beddington, 2010), and of biological invasions (Venette et al., 2010) on the future impacts of pests and diseases on agricultural systems.

In part because crop pests and diseases are inherently part of cultivated systems, the measurement of their impact on crop performances is a field of its own (e.g., Madden, 1983, Campbell and Neher, 1994, Brown and Keane, 1997, Savary et al., 2006). Only some overall estimates are available, among which is the often cited ranges produced by Oerke (2006). Esker et al. (2012) provide a recent review of the current scientific framework to assess the importance of pests and diseases to crop production, including consideration (i) of production situations and associated (uninjured) attainable crop yields, (ii) of the effects of yield-limiting factors (i.e., abiotic stresses) on the harmful effects of pests and diseases, and (iii) of the interactions among pests and diseases. These three elements have been analysed in a few important crop-pest systems, such as in potato in the USA (Johnson, 1992), groundnut in West Africa (Savary et al., 1990, Savary and Zadoks, 1992), lowland rice in tropical Asia (Savary et al., 2000a, Savary et al., 2000b), and wheat in Western Europe (Willocquet et al., 2008). These examples indicate that (1) the impact of pests and diseases may strongly depend on production situations and on the associated attainable yields; (2) ignoring the interaction of pests and diseases may lead to substantially incorrect estimates of their impact on agricultural production.

The improvement and application of PDM for predicting yield losses to reduce risks to global food security and adaptation to climate change is still a challenge for the scientific community (e.g., Garrett et al., 2006, Savary et al., 2011). Data collected in various environments no longer represents a reference data set for the development of empirical models, because the climatic patterns the models were calibrated for are changing. Because it enables addressing ‘what if’ questions on the basis of quantitatively known processes, simulation modelling represents a central approach to estimating the impact of the potential effects of climate change on agricultural production.

The objective of this paper is to present an analysis of the technical and scientific challenges in the development of process-based models for pest and disease modelling, and a possible road map to improve their capability for estimating impacts on agricultural production.

## 2 New challenges and goals

Applied modelling of crop diseases and pests has been dominated by short term, tactical questions, such as the development of support capabilities to schedule scouting or pesticide applications, i.e., decision support systems (DSSs; e.g. Welch et al., 1978, Magarey et al., 2002, Isard et al., 2015). These modelling activities are often based on specific pest-crop systems, in specific environments, and based on multi seasonal observations, that allowed the building of robust empirical relationships using weather variables and crop phenology (Madden et al., 2007). Working on given, local patterns of weather variation and on specific pathogen and pest species has simplified the representation of the interactions between a biotic stressor and a host. Key aspects in the development of DSSs include knowledge on system dynamics, built on data from multiple seasons and collected in the pest-crop systems of interest (Madden et al., 2007). An alternative approach has been to build models parameterized from independent, controlled experiments, targeted at identifying organisms responses to a range of environmental factors. Two of the most popular examples are phenology models for insect pests (Welch et al., 1978) and SEIR (Susceptible-Exposed-Infectious-Removed) and infection models for plant pathogens (Zadoks, 1971, Magarey et al., 2005). These kinds of models could have application for determining how the changing climate might also alter the frequency of pesticide applications. In some cases, it may be possible to estimate yield impacts by converting forecasts of pest or disease intensity to projections of yield loss (Dillehay et al., 2005).

New challenges and goals are rerouting or integrating the priorities of pest and disease modelling. The main challenge is due to climate, which has now been demonstrated to change temperature averages, as well as rainfall means and distributions in the season, and to increase their variability. The shift to a non-stationary climate now implies that observed datasets are no longer a sufficient base to predict system behaviour even at specific locations where the data were collected. There is evidence that pathogens which for decades have had no impact on crops in specific environments are now becoming key determinants of crop yield (e.g., Lees and Hilton, 2003, Yang and Navi, 2005, Berger et al., 2007, Parker and Warmund, 2011, Gramaje et al., 2016). At the same time, the increasingly comprehensive goal of estimating risks to global food security requires the inclusion of geographical areas and production system where the available baseline data are not adequate to develop local, robust empirical relationships. Changes in weather patterns make it impossible to address these questions solely via field experiments. Empirical approaches, based on, e.g., statistical models, could rapidly bring about issues associated with non-linearity of responses of processes (Garrett et al., 2006) and for climatic conditions which are beyond the ranges in which models are developed. Also, the goal of making estimates of pest and diseases dynamics under future conditions precludes trend analysis, which would be built on the evidence collected from different weather patterns. Process-based modelling, combined with the careful design of scenarios to analyse impacts, provides an avenue to address these questions. Shared modelling structures among a network of scientists from different fields appear to be a most appealing and efficient way to scientifically address these challenges.

In addition, applications of pest and disease modelling are becoming increasingly important for strategic decisions, such as breeding for host plant resistance in future climate scenarios (e.g., Duveiller et al., 2012), policy-making and priority-setting for research (e.g., Willocquet et al., 2004), applications for risk analysis of alien invasive species (Venette et al., 2010), and for resource allocation (Beddow et al., 2015). A new generation of tools based on state of the art knowledge and technology is needed to allow system analysis including key processes and their dynamics over an appropriate range of environmental variables.

## 3 Modelling approaches and perspectives

The dynamics of plant diseases and pests and the processes involved in crop growth and crop performance injured by pests and diseases correspond to two distinct sets of processes. These processes have traditionally been studied by different scientific communities, leading to a wealth of knowledge, which can be mobilized to address questions related to the impacts of pest and diseases on crops. However, attempts to couple PDM to crop models may have led to over-simplifications either of the crop, or of the pest or disease. Alternately, very detailed crop models are very hard to link to highly detailed disease or pest models. A first objective is to couple state of the art modelling knowledge for each of the different communities. A second objective is to define clear modelling objectives, which lead to transparent decisions with respect to the level of detail required in models.

### 3.1 Model type and purpose

A broadly accepted view (Savary et al., 2006, Esker et al., 2012) is that *injuries* caused by harmful organisms (diseases and animal pests) lead to *damage* (i.e., to crop organs), and that damage leads to (yield) *losses*. The three elements, injury, damage, loss, are linked by two relationships (Zadoks, 1987): a damage function translates injury into damage (crop losses), and a loss function translates injury into economic loss. Much work has addressed the shape of the damage function: depending on the considered system, the damage function depends on the production situation (Rabbinge et al., 1989, Savary, 2014), on the genotype of the host, or on the interaction with other harmful organisms (Zadoks, 1985, Savary et al., 2006). The modelling of the damage function has been undertaken using a range of approaches. Statistical approaches, in particular (Campbell and Madden, 1990, Esker et al., 2012), have contributed to show that a system approach was useful, not only to predict but also to understand crop losses: the number of factors that may affect the damage function can be large.

**Fig. 1:**
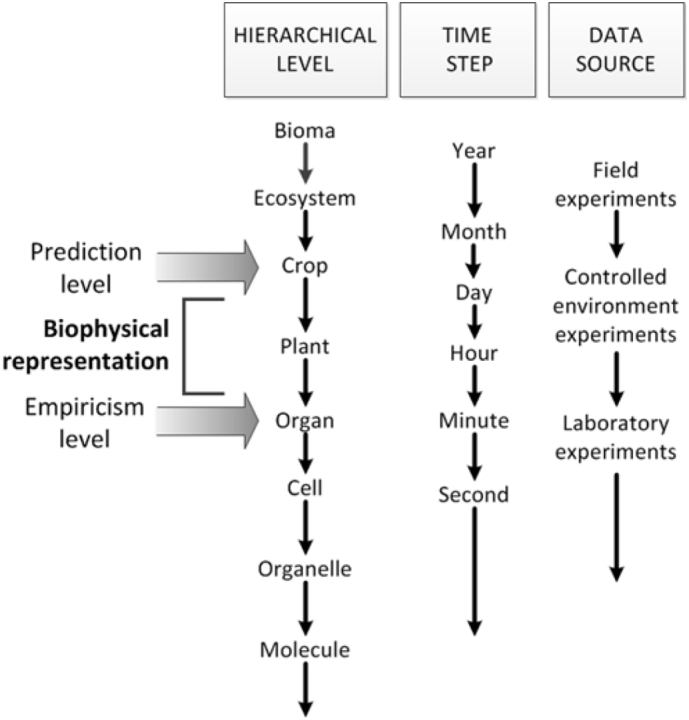
Prediction and empiricism levels in process-based crop simulation models. Redrawn from Acock and Acock (1991).

However, becoming aware of the existence of factors and their interaction does not mean that the empirical relationships can be used when considering yet-to-exist contexts. Similar to the analysis made for crop models on levels of empiricism (Acock and Acock, 1991) and represented by Fig. 1, building process-based models implies making predictions two or three levels above the one where the empiricism is built; parameters should have a biological meaning and the construct will be a hierarchical representation based on system analysis. Such analyses have been done for many models, and the approaches chosen to simulate each process needs to be reconsidered with regards to the interactions with biotic constraints.

### 3.2 Current trends in pest and disease modelling

Several reviews (e.g., Savary et al., 2006, Esker et al., 2012) have documented recent advances made in the field of designing generic simulation models for pest and disease, and for crop losses. Process-based modelling appears to be a critical approach to quantitatively address questions pertaining to the behaviour of complex systems, such as the crop-pest and pathogen systems. A first challenge to consider is the diversity of pests and diseases that affects cultivated crops, including arthropods, nematodes, fungi, oomycetes, bacteria, viruses, and mycoplasma. We summarize below a typical approach in plant disease epidemiology for disease process models, which provides guidance:

1. The disease cycle is represented by an infection chain (Kranz, 1974), which becomes the focus of analysis;
2. Each step of the infection chain corresponds to a functional trait (Pariaud et al., 2009) of a given pathogen in a particular pathosystem;
3. Each functional trait leads to quantifiable processes, that can be analysed in terms of efficiency and performance, especially in response to environmental factors, including the host and the biological environment (Zadoks and Schein, 1979);
4. The resulting process-based information on each process constitute the building blocks of a simple, generic, process-based modelling structures (e.g., Savary and Willocquet, 2014, Bregaglio and Donatelli, 2015).

**Fig. 2:**
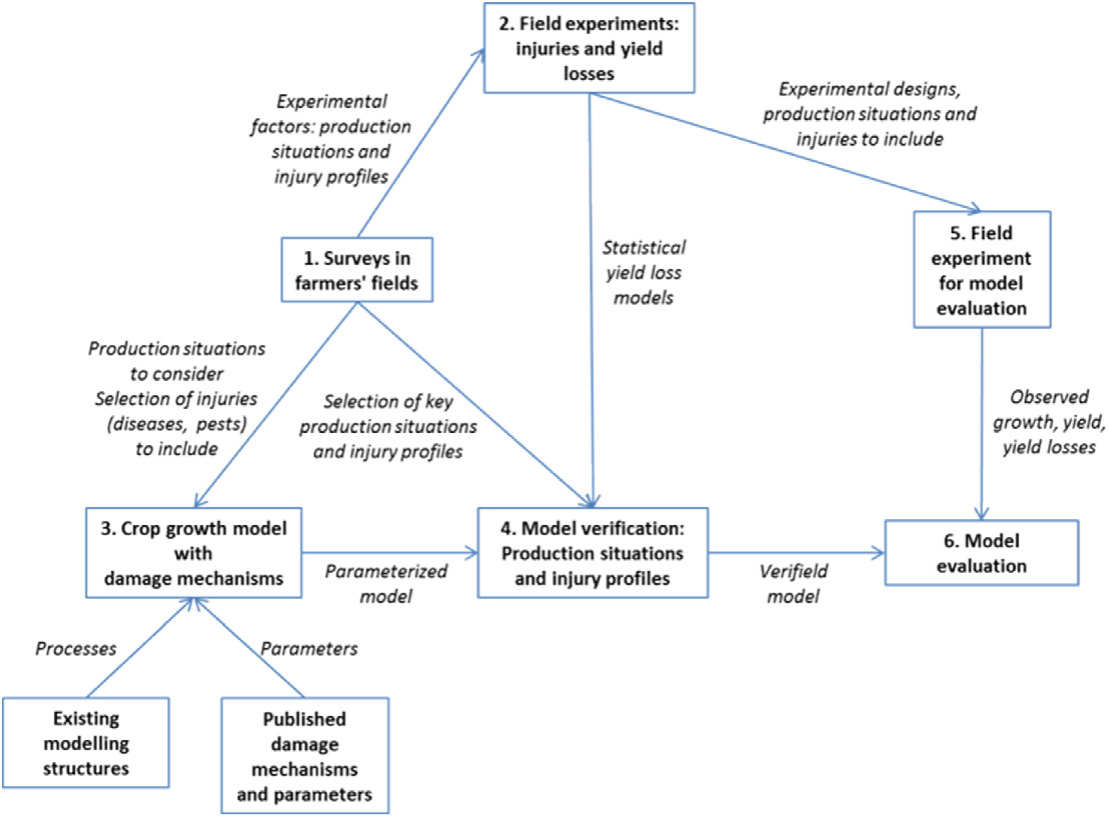
A summary flowchart of steps involved in the modelling of crop – pathogen and pest systems.

Plant pathologists have developed a large number of such disease models modelling structures, where the emphasis is placed on the mobilization of primary inoculum, the production, spread, and efficiency of secondary inoculum, or both (e.g., Rossi et al., 2009). As for crops, there are well-established modelling platforms, as cited in the introduction, which target the simulation of the interaction genotype x environment x management (Fig. 2).

A second challenge corresponds to the variety of interactions that may exist between pests and pathogens, and the growing crops. As discussed in Savary et al. (2006), a range of concepts have made the modelling of crop-pest and disease interactions possible through generic, mechanistic, agrophysiology-based simulation. The diversity of harmful organisms to crops (pathogens, animal pests, and weeds) can be captured in a small number of guilds, each corresponding to one type of injury mechanism (Rabbinge and Rijsdijk, 1981, Boote et al., 1983). Thus, process-based agrophysiological models can be used to simulate yield losses (Rabbinge et al., 1989, Rouse, 1988). Modifiers (Loomis and Adams, 1983) can also be used to represent reduced performance at specified points of the modelling representation of the system. Building upon Monteith's (1972) simplified approach of crop growth, injuries have also been pooled in two main groups: intercepted radiation or radiation use efficiency reducers (Johnson, 1987, Waggoner and Berger, 1987).

One option to formalize models is via generic simulators. Generic simulators identify key processes to represent living organisms which are abstracted to functions whose parameters allow the representation of different species. New functions can be added to extend the application of generic simulators to species that have more specialized biology. Consequently, once a generic simulator is developed, less resources and time are needed to develop a species-specific model, mostly via parameterization; this avoids duplication, facilitates maintenance, and makes comparison of modelling approaches simpler. Another added value is that a generic framework serves as a template for the collection of the required biological information for such an activity. For arthropod plant pests, generic models have been developed for insect phenology (Welch et al., 1978), insect populations (Shaffer and Gold, 1985, Yonow et al., 2004) and non-indigenous pest development (Sutherst et al., 2007, Hong et al., 2015). They require only a few parameters and a minimum set of input data. The template approach to modelling has been successfully used for soil conservation (Steiner et al., 2006), for agricultural crops (Wang et al., 2002, Jones et al., 2003), and for arthropod pests and diseases (Sutherst et al., 2007, Manici et al., 2014, Bregaglio and Donatelli, 2015, Magarey et al., 2015).

The progress and impact of modelling work is greatly enhanced when models can be shared and modified among a broad scientific community. For example, in genomics, synteny analyses produces analytical results far beyond that which could be expected from the informal aggregation of fragmented results (Tatusov et al., 2000, Stein et al., 2002). A recent example of knowledge sharing in biophysical modelling is represented by AgMIP (Agricultural Model Intercomparison and Improvement Project), a major international collaborative effort to assess the state of global agricultural modelling and to understand climate impacts on world agriculture (Rosenzweig et al., 2013). To the best of our knowledge, examples of formal modelling networks shared and used by a scientific community do not exist within the crop health disciplines. In the case of plant disease, process-based models of the SEIR type may represent a valid entry point for a generic modelling effort. This type of model is generic even beyond the field of agriculture, since the basic concept is also broadly used in animal (van der Goot et al., 2005) and human disease epidemiology (Newton and Reiter, 1992). The processes accounted for by this model type indeed capture epidemiological processes that govern epidemic build-up: disease transmission, delay between infection and infectiousness of the host. Concepts and theories that exist and have been applied in a fragmented way so far can therefore be mobilized towards an effort for a generic epidemiological modelling platform. An illustration of the genericity and applicability of SEIR models for plant disease has been recently made available online on the APSnet Plant Health Instructor (Savary and Willocquet, 2014) and an extensible simulation package (Bregaglio and Donatelli, 2015). The SEIR type models typically consider two levels of hierarchy: (1) monocyclic processes, i.e., infection, latency, sporulation in the case of aerially-dispersed pathogens, and (2) the epidemic process, i.e., the dynamics of disease in a population of plant hosts. Monocyclic processes can be influenced by environmental factors such as temperature and moisture, which can be used as model climatic drivers. Simulated epidemics can be represented for example by the number of lesions per crop unit area. These simulated outputs can in turn be used as inputs for crop models that account for damage mechanisms, i.e., the physiological effects of disease on crop growth and yield (Rouse, 1988). Epidemiological models can therefore be linked to crop growth models to simulate yield losses caused by diseases. Crop growth models that include damage mechanisms have been developed over the last decades (e.g., Bastiaans et al., 1994, Pavan and Fernandes, 2009), using the concept of “coupling points” (Boote et al., 1983). Although these models were developed by different teams, on different crops, they were all grounded on the generic concept of damage mechanisms, which can be applied not only to a range of diseases, but also to the other yield-reducing factors (e.g., insects and weeds). GENEPEST, a generic crop growth model including the damage mechanisms of pests, has been recently made available online on the APSnet Plant Health Instructor (Savary and Willocquet, 2014); the framework *Diseases* in BioMA (Donatelli et al., 2014b) includes a module for the damage on plants and a module to simulate the impact of diseases control via agricultural management (Bregaglio and Donatelli, 2015).

### 3.3 Data requirements

Common inputs for PDM are air temperature, precipitation, relative humidity, and leaf wetness (Magarey et al., 2001), at daily or hourly resolution. Other variables such as soil temperature, radiation, wind speed, and direction are used in more specialized models such as those targeting aerial transport or soil pathogens. For many PDM, daily weather data is sufficient, but for many disease models hourly data is required, which can be estimated for scenario analysis with an acceptable level of accuracy (e.g., air relative humidity, Bregaglio et al., 2010). Additionally, numerical weather models can provide gridded data at increasingly finer spatial resolutions, both for current and forecasted data (three-fifteen days). Examples of gridded datasets that can be used for plant disease forecasting include the Real Time Mesoscale Analysis system (RTMA) in the United States (De Pondeca et al., 2011) and AGRI4CAST in Europe (JRC, 2015), and Climate Forecast System Reanalysis (CFSR ) globally (Saha et al., 2014). Many of these datasets have ten or more years of historical data, allowing researchers to conduct simulations in the past. For plant disease forecasting, leaf wetness has been a limitation since the data has historically not been collected by weather stations, except those specifically deployed for agricultural monitoring or for research. However, the use of simulation models is now proving to be a practical alternative (Magarey et al., 2006, Bregaglio et al., 2012). When targeting scenarios of climate change, assumptions need to be made for weather variables which are not a direct output of global circulation models, such as wind and relative humidity.

When coupling PDM to crop models with the aim of developing an operational tool for pest and disease management, the limiting factor is often the lack of ad-hoc benchmark datasets. Many PDM also require other agronomic inputs such as the leaf area index, the height of the canopy, the width between canopy rows (or other measures of foliage density) and soil type (e.g., Batchelor et al., 1991). However, model evaluation generally requires datasets built on experiments which are designed to contrast treatments to minimize the risk of making a data-fitting exercise when performing calibration, as discussed in the next section. Such contrasting treatments might not make any agronomic sense and consequently are in general not available in field experiments. Considering the coupling of PDM to crop models to estimate the impact on yield, both models need to be verified. This would require specific field trials, where the crop is grown in optimal water and nitrogen conditions, both factorially crossed with at least two levels of disease and pest injuries: “absent” and “present” (Esker et al., 2012). This articulated design is actually not sufficient when multiple disease and pests are addressed. In such a case, very large, multi-season field experiments are to be considered (e.g., Savary et al., 2000a, Savary et al., 2000b). Such experimental designs however are at the base of model evaluation with the aim of providing guidance in identifying causes of the mismatch between model predictions and the real system performance. These experiments are costly, but the evaluation of coupled pest, diseases and crop models must be thoroughly performed to build confidence in their predictive capabilities, while contributing to the general understanding of system behaviour. Other datasets, with a lower level of detail, can be collected from actual fields to corroborate the model development and calibration made with the detailed dataset presented above. This is described in greater detail in the section on a roadmap to improve pest modelling.

### 3.4 Model calibration and evaluation

The term calibration is overloaded in the scientific community. Limiting the discussion to process-based crop models, model users quite often use the term calibration for all actions related to assigning parameter values, both to those which have a biophysical meaning (e.g., maximum specific leaf area), and to those which are more summary parameters to account for factors that are not considered in a specific modelling approach (e.g., empirical coefficients to modulate growth and maintenance respiration). However, the difference in handling these two groups of parameters is substantial. In one case the values must have a biophysical meaning, often resulting from physical experiments; in the other case they can be adjusted iteratively by minimizing a cost function. In the latter case, a model that requires such optimizations to explain a substantial part of the mismatch between simulated and observed values cannot be used outside the specific conditions used for calibration.

Many disease and pest models are parameterized from experiments conducted under controlled environmental conditions. For example, many experiments measure development time of insects (total and stage specific), mortality, fecundity and longevity under different temperatures (Regniere et al., 2012). These data can be used to parameterize a variety of models including phenology models based on thermal time and population models that predict the proportion of individuals in each life stage and the total population. Likewise, experiments where plants are inoculated under different temperature and wetness regimes can be used to parameterize infection models (Madden and Ellis, 1988, Magarey et al., 2005). There have been a few efforts to compile parameter libraries, collecting developmental data including thresholds and degree day requirements for insects (Nietschke et al., 2007, Jarošík et al., 2011) and infection requirements for pathogens (Magarey et al., 2005). A common approach when data for a given species is lacking is to identify parameters from closely related species. In this case, field studies may also be helpful when controlled data are absent, particularly by allowing a modeler to see if estimated parameters fit observed data.

PDM evaluation is essential since it allows the modeler to know if the simulations are in line with the real system. There are several ways models are currently evaluated in plant pathology and entomology (e.g., Rabbinge, 1993). This includes comparing simulations against observed pest and disease intensity in, for example, sprayed and unsprayed plots. In plant pathology and entomology, model evaluation is usually done by the same parties that developed the model. An important issue is the risk of overfitting, i.e., when parameters of the model are adjusted until the model output matches very closely to the training data, and the same model shows poor performances when applied on independent datasets. Overfitting thus leads to false confidence in a model's accuracy and even to failure in conditions that do not exactly match those of the training data.

The robustness of a model can be estimated from the stability of performance across treatments and environmental conditions; overfitting using datasets that poorly represent environmental conditions and potential vs. actual management, results in a model with low robustness (Bellocchi et al., 2010). Estimating the applicability of a model to new conditions is qualitative, and has two requirements: i) an estimate of robustness as result of model evaluation, and ii) the evaluation of model structure (also evaluating the level of empiricism) compared to the major performance drivers of system, to verify that the model accounts for the relevant processes. Robustness and evaluation of model applicability are even more critical when considering the coupling of PDM to crop models.

## 4 Modelling frameworks

The generic term modelling framework may refer either to conceptual workflows for model development and/or to actual software realizations to develop and run modelling solutions (Holzworth et al., 2015). The main desirable features of a modelling framework are extensibility of modelling approaches and modelling solutions, transparency, and the capability to interface to various sources of data. These features allow easier model comparison and model evaluation against a larger number of datasets compared to what can be done with separate model tools. Modelling frameworks may also facilitate model construction, allowing a more direct link to the results of research (Donatelli et al., 2014a) by enabling the easier use of new findings in existing modelling solutions.

The following examples are not exhaustive and represent different typologies of modelling approaches and tools available to simulate pest and/or diseases epidemics and impacts on crops.

The modelling of biotic injuries over time and space is a well-established field of its own, with different names depending on the scientific areas: plant disease epidemiology (or botanical epidemiology) in plant pathology, and population dynamics and ecology in the animal sciences, for instance. Grouping these different fields into a single modelling framework is probably neither possible, nor desirable – the modelling of population dynamics for instance addresses themes of their own, such as population biology, plant-pest coevolution – which do not necessarily overlap with the harmfulness of agricultural pests. We therefore focus here on the key issue of the inclusion of disease and pest impacts in the modelling of crop growth and crop performance.

Crop and cropping system modelling is nowadays often represented by platforms, some of which have evolved over more than 20 years. They may consist of generic crop simulators such as CropSyst (Stöckle et al., 2003) or STICS (Brisson et al., 2003), or of platforms which share parts of the simulation engine (i.e., modelling approaches) and retain specific modules for crops, such as DSSAT (Jones et al., 2003) or APSIM (Brown et al., 2014). Fruit tree crop models are specific for species (e.g., Lakso and Johnson, 1990, Grossman and De Jong, 1994). These models may include modules to account for the damage due to biotic stressors, but these modules are embedded into the code.

### 4.1 APSnet

On the American Phytopathology Society website (APSnet), an educational module on Simulation Modelling in Botanical Epidemiology and Crop Loss Analysis provides an overview of PDM and crop loss models. It also includes an introduction to a number of generic models including the GENEPEST model, as well as instructions for running the models. An overall framework for modelling the impacts of pest and diseases on agricultural systems using these kinds of models is provided in Savary et al. (2006), which we can summarize as follows:

1. farmers' fields surveys are conducted over a given geographical range, at many locations, and several years, to characterize (i) production situations (PS) and (ii) injury profiles (IP);
2. field experiments are conducted to measure and statistically model PS, IP, and PS x IP effects on attainable yield (Ya), actual yield (Y), and yield losses (Ya - Y);
3. a mechanistic simulation model of crop growth and yield is built, to account for (i) features of PS influencing crop growth (yield defining and yield limiting factors), and (ii) processes which may be affected by damage mechanisms;
4. this preliminary model for crop growth, yield accumulation, and yield reduction is verified through a series of evaluations involving (i) a range of parameters that account for the characterized production situations (effects on attainable yield, Ya), and (ii) a range of levels of injuries derived from the injury profiles characterized during farmers' field surveys;
5. a series of field experiments are conducted at several locations, in a range of climatic conditions, and at different levels of input, in order to mimic varying production situations, and with a range of levels of injuries, corresponding to the injury profiles characterized in farmers' fields;
6. simulation outputs are confronted to results from field experiments to assess the ability of simulations to account for (i) effect of production situations (PS) on attainable yields, (ii) effects of individual injuries and injury profiles (IP) to reduce yield from attainable to actual, and (iii) PS x IP interactions on crop growth and yield.

This approach has been followed in the case of the rice-multiple pest system in Asia, where the successive steps above have been documented (Savary et al., 2000a, Savary et al., 2000b, Willocquet et al., 2000, Willocquet et al., 2002, Willocquet et al., 2004). It also has been implemented in the case of the wheat-multiple pest system in Western Europe, using extensive, published survey work in the Netherlands (Daamen, 1990, Daamen and Stol, 1990, Daamen and Stol, 1992, Daamen and Stol, 1994, Daamen et al., 1991, Daamen et al., 1992), and the UK (King, 1977, Polley and Thomas, 1991, Foster et al., 2004), as well as a large body of published parameters on damage mechanisms in the wheat – multiple pest system (Willocquet et al., 2008).

Fig. 2 sketches the relationships between the six stages presented above. Variation may of course occur depending on the crop – disease and pest system considered, however Fig. 2 emphasizes the importance of field work: farmers' field survey, which produce the essential information on production situations and injury profiles, and field experiments with a design specifically developed for modelling purposes.

### 4.2 The APSIM-DYMEX link

The Agricultural Production Systems Simulator (APSIM), is a systems modelling framework that has been developed over the last 20 years (Holzworth et al., 2015). The collection of models available within APSIM provide tools and resources to explore the dynamics of agricultural landscapes. APSIM does not incorporate pests and diseases. Some work examining competition between weeds and crops has occurred (Deen et al., 2003, Robertson et al., 2001), which was extended to modelling of the weed seed bank (Smith et al., 2000) and genetic dispersal of resistant weeds (Thornby and Walker, 2009). However, limitations within these approaches prevented further development (Whish et al., 2015a). A recent addition to APSIM has been the linking of the population modelling framework DYMEX (Whish et al., 2015a). DYMEX (Sutherst and Maywald, 1998) was developed to simplify the construction of mechanistic, process-based population models (Sutherst et al., 2000) and has been used to describe the life cycles of insects, weeds and diseases. Models are constructed within the DYMEX building software and compiled to run within the DYMEX simulator. The linking of DYMEX and APSIM was favoured over the construction of a specific pest and disease module within APSIM because it reduced overheads and capitalised on the history and success of both modelling frameworks (Whish et al., 2015a). The link between the two frameworks was created by wrapping the DYMEX simulation engine within APSIM. This approach took advantage of the multi-point features within APSIM (the ability to simultaneously simulate multiple points in space and the interactions between them) and the input/output features that simplified communication between multiple models. The integration of DYMEX as an APSIM component allows the DYMEX component to execute with the rest of the APSIM simulation, accepting information from other modules (e.g. weather data from APSIM climate files or soil moisture from the water balance model) and sending information (population size, infected leaf area) to other models within the APSIM framework. The use of the generic wrapper to link the two frameworks, allows any model constructed in the DYMEX building tool to run within APSIM. The DYMEX-APSIM link has been successfully used to model rust (*Puccinia striiformis*) growth on wheat and demonstrated the interactions of large rust populations reducing the wheat crops leaf area (Whish et al., 2015a). An examination of the population decline in root lesion nematodes (*Pratylenchus thornei*) over a non-host fallow is another example of this approach (Whish et al., 2015b).

### 4.3 NAPPFAST

An example of the interactive modelling templates was the North Carolina State University/Animal and Plant Health Inspection Service Plant Pest Forecasting System (NAPPFAST; Magarey et al., 2007, Magarey et al., 2015) that was an active project between 2002 and 2013. The NAPPFAST system employed an internet-based graphical user interface to link interactive templates with weather databases. NAPPFAST included three modelling templates: a degree day template for creating phenology models for arthropod pests and plants, an infection model template for plant pathogens, and a generic template for creating simple empirical models; e.g., hot and cold exclusion. Each template follows a simple fill-in-the blank design. All templates in NAPPFAST were generic (i.e., applicable to many species) to meet the needs of diverse users. The templates in NAPPFAST were linked to stations and to North American and global gridded weather databases. The capabilities allowed NAPPFAST to create pest risk maps (Magarey et al., 2011) at resolutions of 5 km in the United States and 38 km globally. More recently some of the technologies developed for NAPPFAST have been applied for the integrated Pest information Platform (iPiPE) project. The iPiPE was created to promote the exchange of pest data among agricultural professionals (Isard et al., 2015). It is an information technology platform that provides tools and models for managing and analyzing data in order to generate products and commentary for integrated pest management (IPM) and national food security. The iPiPE brings together Extension professionals, county agents, crop consultants, industry, federal, and state partners by allowing the exchange of pest observations while protecting client privacy. Like NAPPFAST, the iPiPE will include models for simulated pest phenology, infection and pest intensity (Hong et al., 2015) but will use hourly weather inputs. Although the models available in the iPiPE are designed to primarily simulate the timing of pest occurrence to enable management operations (such as scouting) the modelling approach could potentially be used to estimate the impacts of pests. For example, impacts might be estimated from simulations of pest or disease intensity in combination with estimates of host phenological susceptibility (Dillehay et al., 2005).

### 4.4 BioMA-Diseases

**Fig. 3:**
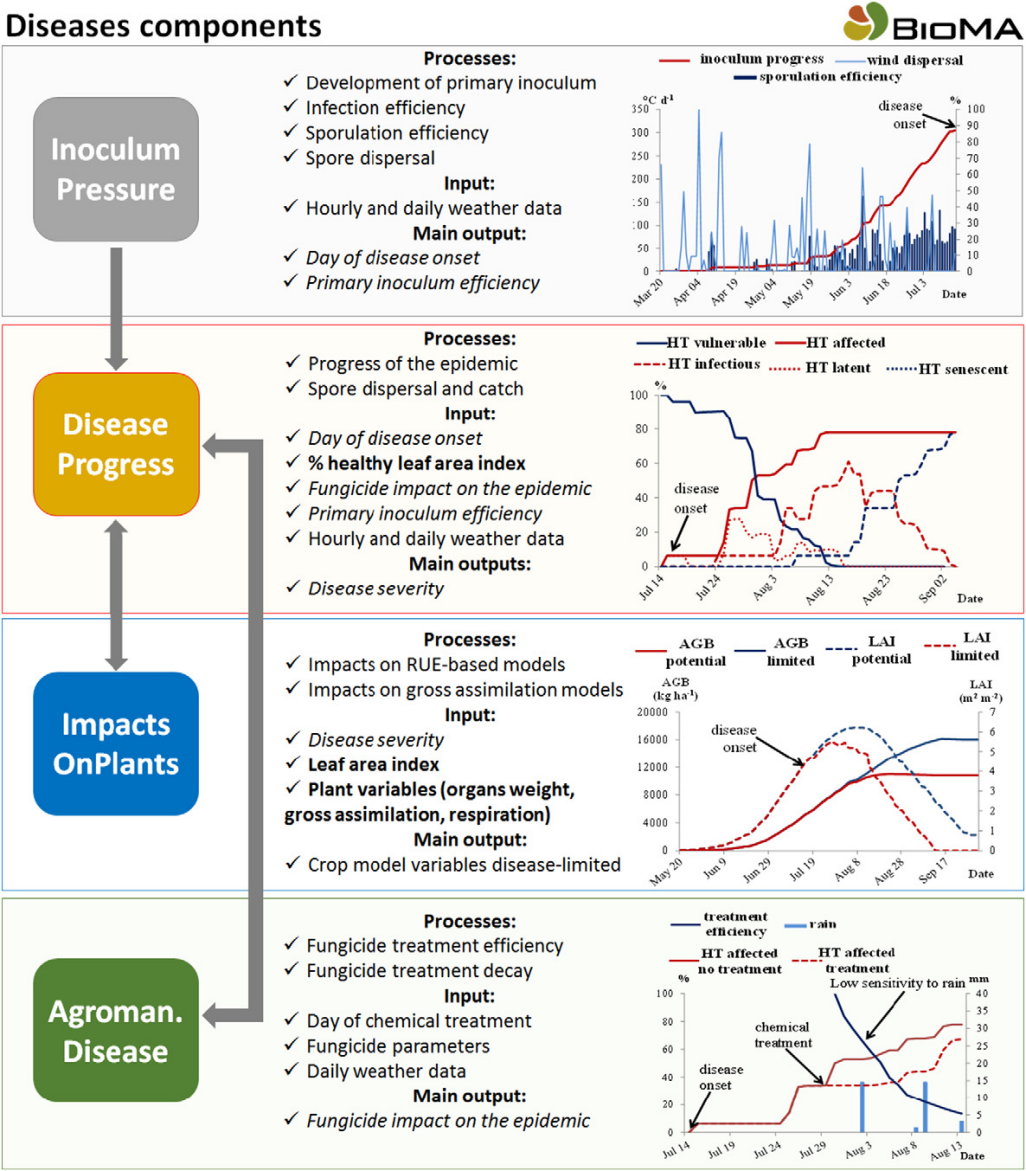
Schematic representation of the four Diseases components (coloured boxes) and of their interaction (grey arrows). For each component, the main processes, inputs and outputs are reported, with charts presenting sample simulations. The variables shared among Diseases components are reported in italics; the variables produced by the crop model are reported in bold. HT = host tissue, AGB = aboveground biomass, LAI = leaf area index.

This modelling framework (Bregaglio and Donatelli, 2015) is composed by four extensible software libraries targeting the modelling of a generic fungal plant diseases. It provides input/output data structures and models to simulate a polycyclic fungal plant epidemic and to quantify its impact on crop growth. The rationale guiding the development of this framework entails the definition of four sub-domains in the modelling of plant disease epidemics: (i) the production of primary inoculum and the occurrence of primary infections, (ii) the development of secondary infection cycles during the cropping season, (iii) the interactions between epidemic development and crop physiological processes and (iv) the impact of agricultural management practices on disease development (Fig. 3). This discretization also reflected in the software development of the components, which provide users with an existing definition of specific domains to focus on when introducing new models, other than favouring their stand-alone application and extension. These tools were developed according to the specifications of the BioMA framework, which is a public domain software framework designed and implemented for developing, parameterizing and running modelling solutions based on biophysical models in the domains of agriculture and environment (Donatelli et al., 2014b). The adoption of component-oriented programming and the definition of the ontology of input and output variables promote the link of the *Diseases* components with large area databases and their interface with external tools to perform model sensitivity analysis. Two applications of this technique were realized on major diseases of wheat (brown rust) and rice (leaf blast) in Europe and China, respectively, to test model behaviour under heterogeneous weather conditions according to changes in parameters values. Although the main target of the *Diseases* component is the scenario assessment when limited reference data are available (e.g, in climate change conditions), a recent study by Bregaglio et al. (2016) documents the extension and application of *Diseases-*based modelling solutions to reproduce reference field data referred to the annual fluctuations of rice blast disease epidemics in Northern Italy.

## 5 A roadmap to improve pests and diseases impact modelling

**Fig. 4:**
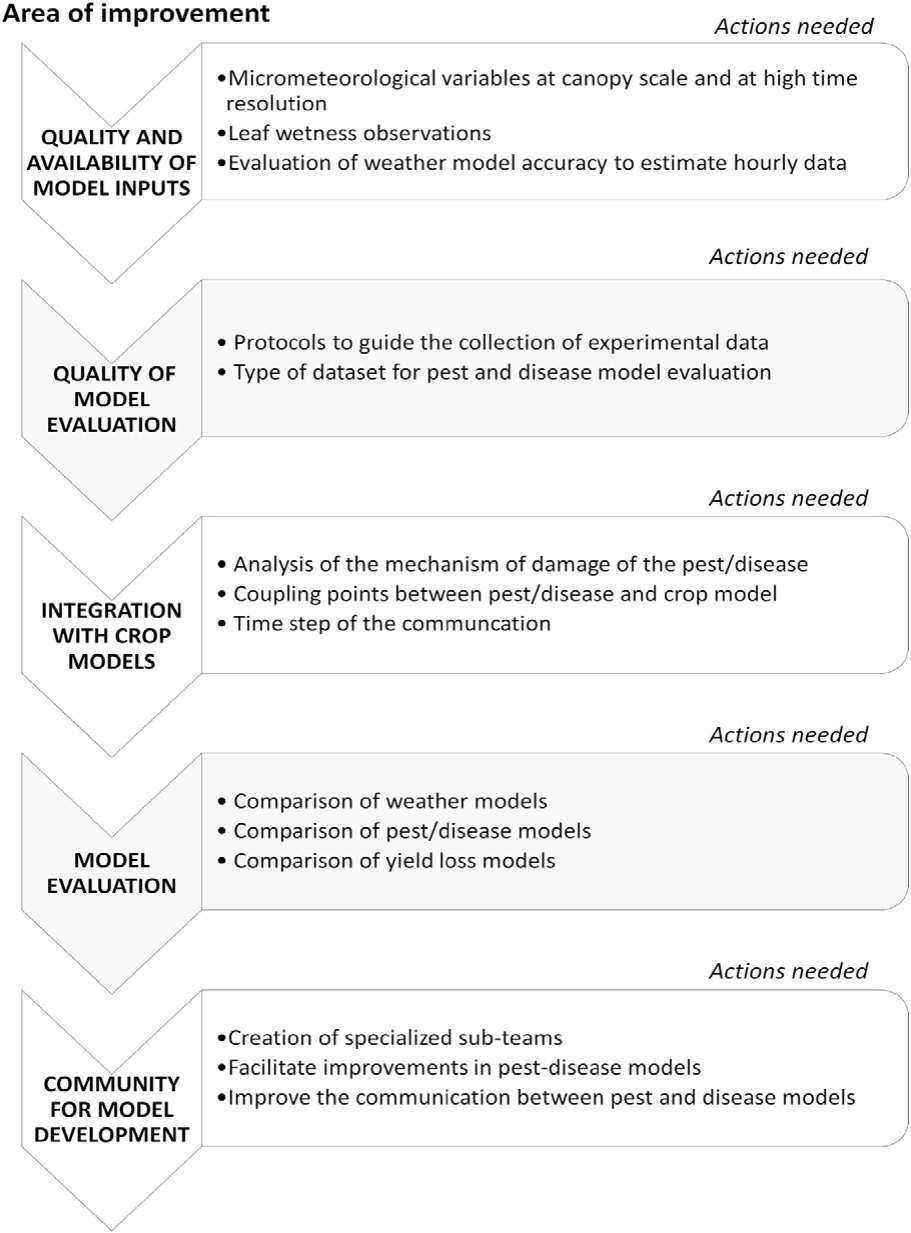
A roadmap for pest-disease-crop integrated model development

We propose a roadmap to improve the simulation of the impacts of pests and diseases in agricultural crop simulation models. The action plan concerns five areas: i) improve the quality and availability of data for model inputs; ii) improve the quality and availability of data for model evaluation; iii) improve the integration with crop models; iv) improve the processes for model evaluation and v) develop a community of plant pest and disease modelers (Fig. 4).

### 5.1 i) Improve the quality and availability of data for model inputs

The process-based modelling of the dynamics of plant pests and diseases aims at reproducing the biophysical processes guiding their development and spread in time. The effect of weather conditions has traditionally been an important focus of these models. The dependency of the pathogen growth rates of pathogens on the variability of weather conditions implies that models should reproduce these relationships by modulating their responses accordingly (Magarey et al., 2005, Pfender et al., 2012). The availability of high-quality input datasets is necessary to calibrate PDM parameters, for instance the ones related to temperature and moisture response functions. As discussed in the section above, the main drawback of low-quality datasets is the reduction of model parameters that have a biophysical meaning to empirical coefficients that merely improve model fit to reference data. This is why the quality of input data is key in pest and disease modelling: micrometeorological variables at canopy scale and at high time resolution, such as air temperature, relative humidity and leaf wetness are needed to reduce the uncertainties during calibration and evaluation activities. In particular, the availability of leaf wetness observations for pest and disease forecasting/modelling is often limited to specific experimental trials, being constrained by the presence of leaf wetness sensors on agricultural weather stations (Lee et al., 2015). For this reason, a viable alternative for leaf wetness data to drive PDM on large scales is the estimation of leaf wetness from commonly measured meteorological variables (e.g., Magarey et al., 2005). Leaf wetness simulation models have been developed since 1982 to estimate leaf wetness (e.g., Magarey et al., 2005, Sentelhas et al., 2006), but more effort is needed to evaluate their reliability under a range of weather conditions and cropping systems (Bregaglio et al., 2011). For example, gridded numerical models are now able to supply weather information on hourly basis and at a 5 km resolution in the United States (De Pondeca et al., 2011). However, this information has to be downscaled to the level of a canopy to provide accurate pest disease forecasts. Defining the limits of applicability in pest and disease modelling studies is necessary when they are applied under unknown temperature and wetness regimes, as in the case of climate change studies.

### 5.2 ii) Improve the quality and availability of data for model evaluation

Although field observations of pest and disease impacts on crops have been widely collected for many years (e.g., Nutter, 1989, Esker et al., 2012), measurement methods lack standardization, and usually are not linked with weather or agronomic data to enable their use as inputs for PDM. As a consequence, the extensive validation of PDM across diverse environments has been limited to very few cases (e.g., Willocquet et al., 2000, Willocquet et al., 2002, Willocquet et al., 2004). Consequently, there is a need to design protocols which can guide the collection of the experimental data needed to calibrate and evaluate PDM and crop loss models, including both epidemiological and crop data (see e.g., Willocquet et al., 2000), as summarized in the section Data requirements. We propose here a tentative distinction between high (HQ) and medium (MQ) quality reference datasets for model calibration and evaluation, according to the typology of the variables to be measured and to the frequency of their sampling during the growing season.

A HQ datasets for PDM calibration and evaluation should include the full complement of data, including injury measurements, environmental (weather), and agronomic (crop growth and development) data characterizing the impact on the crop. Experimental observations should include multiple measurements of pest and disease injuries (e.g., severity or incidence depending on the injury) during the growing season, and the quantification of yield loss due to pests and diseases. Additionally, detailed measurements related to plant physiological processes as affected by the pathogens should be performed, including for instance effects on photosynthesis, maintenance respiration and leaf senescence. Injury assessments should be collected in unsprayed experimental plots as well as on protected plots (Zadoks and Schein, 1979, Savary et al., 2006, Esker et al., 2012). Weather data should include temperature, relative humidity, precipitation and leaf wetness (whenever appropriate, e.g., in the case of diseases of the foliage). Reference leaf wetness data, for instance, should be collected either using visual observations or a camera at a limited number of sites. Agronomic observations should include attainable (i.e., uninjured) and actual (injured) yield data, as well as leaf area index, crop height, variety, previous crop, and pesticide applications. MQ datasets would not include dynamic information collected during the growing season, but must include quantitative information to characterize the level(s) of injuries (e.g., severity or incidence at key crop development stages) and their impact on crop performance (final yield), other than basic meteorological data to drive PDM.

### 5.3 iii) Improve the integration with crop models

The dynamic linkage between disease and pest injuries and the host crop is through coupling points between PDM and crop models. The framework presented by Rabbinge and Rijsdijk (1981) and Boote et al. (1983) describes seven mechanisms of pest and disease damage on crops - i.e., light stealer, leaf senescence accelerator, tissue consumer, stand reducer, photosynthetic rate reducer, turgor reducer and assimilate sappers. Dedicated experiments can be performed to classify and quantify the damage of different pests and pathogens, as done on several pathosystems by Savary et al. (1990), Bastiaans et al. (1994), Bassanezi et al. (2001) and Robert et al. (2006). The translation of these injuries into mathematical functions offers the possibility to incorporate them into the biophysical processes simulated by crop models.

There are examples in literature in which crop and PDM are linked in different ways, ranging from the use of phenological data to initialize the simulation of a disease model (heading date, Del Ponte et al. 2009), to the ex-post application of simulated disease severity on crop model variables (Luo et al., 1997) and to the dynamic integration of PDM and crop model outputs (Pavan and Fernandes, 2009). A pest or a disease can impact crop growth, consequently affecting the resources used by the crop during its life cycle, and having a direct feedback on the system. Also, pests and diseases can be obligated parasites whose life cycle and trophic relationship is driven by the presence of the host. Consequently, in most cases PDM should be synchronously run with crop models. Aside from the modelling knowledge required, this would lead also to potential problems including the complexity of the model architecture, binary incompatibilities when different software platforms are used, difficulties to test such interactive models, and difficulties in sharing such complex models. These issues are addressed by a vast literature, so discussing these aspects is beyond the scope of this paper.

We identify here three main criticalities to be faced when a coupling point is realized:

1. Suitable identification of the damage mechanisms to be considered is necessary to select the crop model outputs to be affected by the pest and disease injuries via coupling points.
2. The outputs of the pest and disease model must be linked to the selected crop model variables, either directly or via additional functions.
3. The time step of the communication between the pest/disease and the crop model must be decided according to the internal time step of the two models.

A simple but efficient classification of crop models identifies two main categories on the basis of the level of detail adopted in the simulation of the accumulation of dry matter (Kropff et al., 1995). The first groups include the most complex models, which upscale the instantaneous CO_2_ leaf assimilation rate at canopy scale, thus simulating the gross photosynthesis and then subtracting the maintenance and growth respiration to achieve net daily growth rate. The models belonging to the other group share the concept of radiation use efficiency, which enables the quantification of dry matter growth rate as a function of the intercepted radiation. Both groups of models produce outputs such as phenological development, leaf area index and daily growth of the different plant organs, usually at a daily time step. The selection of the crop model to be coupled to the pest and disease damage must be done after verifying the presence of the corresponding variable to be affected by the PDM output. For example, if the PDM impacts the increase of crop maintenance respiration, this variable must be an explicit variable of the crop model, otherwise a surrogate variable must be used as a coupling point.

### 5.4 iv) Improve the processes for model evaluation

Improving capabilities to estimate the interaction between pests, diseases and crops requires actions along two lines: building models and modelling tools, and model evaluation. Although we aim at building generic modelling frameworks, model evaluation must focus on specific crops (within crop rotations).

In the AgMIP project (Rosenzweig et al., 2013), a phase of evaluation requires modelers to run simulations corresponding to test data sets for which they have not seen the observations of the response variable (“blind” datasets, for example yield for crop models). For plant pest and disease modelling, one of the evaluation challenges will be to develop appropriate evaluation criteria to judge model success or failure. For example, observations of pest and disease impact may be typically recorded in terms of units such as insect numbers, percentage of host tissue affected or pest incidence. Likewise, PDM may have vastly different output units. It will be necessary to overcome these differences in measurement units in order to statistically compare models performance and to highlight areas for their improvement. This requires the development of standard criteria for model evaluation, which can be tailored to specific crop-pest system and research questions. The definition of such standards will impact the building of datasets, providing specifications on the data model and necessarily leading to metadata definition.

### 5.5 v) Develop a community of plant pest and disease modelers

The development of improved pest and disease models has been hampered by the lack of a cohesive research community. There are several reasons why a community has not developed already. The major point is likely the misunderstanding of roles, in which some modelers might look at experimentalists merely as “data providers.” Likewise experimentalists may under-evaluate the power of modelling tools and consider the abstraction and generalization required for model development as threat to a more detailed biological description of the pest or pathosystem. A special effort which can be acted on building a community as discussed in the coming section is needed to clarify that both model developers and experimentalists are researchers aiming at understanding systems behaviour, and to bridge their communication gap. Another entry point is to increase the community of “modelers-experimentalists”, who implement both skills by conducting the modelling and experimental work in interaction. Another historical limitation is that until recently there have been few generic model frameworks that allowed researchers to move from one pest or pathosystem to another. In addition to what we have discussed above, the limitations of data availability and the absence of standard protocols further limited cooperation in modelling. The Pest and Disease Modelling Intercomparison project (PeDiMiP) was established in 2015, as part of the Agricultural Modelling Intercomparison Project (AgMIP), to address many of the research questions we have outlined in this article. Specifically, the overall goal of PeDiMiP is: "to significantly improve agricultural pest and disease and crop loss models and scientific and technological capabilities for assessing impacts of climate variability, climate change and other driving forces on crop losses, agriculture, food security, and poverty at local to global scales”. To enable this mission, the goal is to create a next-generation knowledge platform for agricultural pest and disease modelling, and coupling it to crop models for worldwide use. Specifically, we propose three objectives i) Improve PDM and their linkages to crop models, ii) Demonstrate the use of PDM for impact assessments, and iii) Create education and training materials for pest and disease and crop loss modelling**.** PeDiMIP is currently composed of three sub-teams, the Crop Health, Potato Late Blight, and Wheat Rust modelling that are working on these objectives.

## 6 Conclusions

The need to estimate the impact of pests and diseases on agricultural production is an important element in the development and analysis of scenarios impacting farmers income and food security. There has a been a shift in the type of model needed to make quantitative estimates of yield loss requiring models with a broader applicability, due both to the need to address the impact of climate change and to the interest on extending the capability of providing estimates globally. To meet both requirements, modelers face the lack of reference data and the need to improve the robustness and applicability of simulation models over such conditions. Historically, obstacles such as the complexity of PDM models and the lack of standards for data collection, model construction, and model evaluation has inhibited the development of both comprehensive modelling tools and a coherent pest and disease modelling community. Although there is a wealth of knowledge on pest and diseases modelling, and on crop modelling in scientific communities, the sharing of knowledge is still quite limited. In this paper, we provide a roadmap for improving agricultural crop simulation models by incorporating the impacts of plant pest and diseases which may be used as a template to address the modelling of a specific pathosystem. We believe that the PeDiMIP and AgMIP projects offer a critical opportunity to overcome these obstacles and so improve the science of cropping system simulation modelling.
